# Enhanced Steering and Drive Adaptations of Modified Ride-On Toy Cars for Improved Directional Control in Very Young Children With Severe Multiple Developmental Impairments

**DOI:** 10.3389/fped.2020.00567

**Published:** 2020-09-08

**Authors:** Juan Aceros, Mary Lundy

**Affiliations:** ^1^School of Engineering, University of North Florida, Jacksonville, FL, United States; ^2^Doctor of Physical Therapy Program, University of North Florida, Jacksonville, FL, United States

**Keywords:** early power mobility, proportional joystick, assistive devices, wheelchairs, modified ride-on cars, child

## Abstract

**Background and Purpose:** One emerging power mobility device (PMD) option that has gained recognition as a means for a cost-effective introduction to power mobility for young children is the battery powered modified ride-on toy car. Many groups, nationally and internationally, have been modifying battery powered ride-on toy cars by adding seating support and a large center placed push button switch for motor activation. The purpose of this technical report is to introduce an enhanced steering and drive system based on proportional control through a joystick.

**Key Points:** This report offers (1) a technical description of these modifications that allow directional steering and programmable starting and driving velocity, (2) an example of a modified ride on toy, including common seating modifications, and (3) a short summary of results from a group of 7 children under the age of six with complex, severe disabilities.

**Clinical Impact:** Although proportional joystick modifications are more complex than the common single switch activation, they allow children greater control to achieve self-initiated, self-directed movement that allow play, peer interaction, and exploration in their natural environments, even in the most highly complex cases. All seven children were able to intentionally self-initiate activation of the modified ride-on car and experience the subsequent movement.

## Introduction

The development of self-guided exploration plays a critical role in the maturation of cognitive, social, emotional and sensorimotor abilities in typically developing children (1). Specifically, as the child explores and engages with their environment by goal-directed, self-initiated locomotion, learning is accelerated, and an understanding of social and spatial relationships enhanced (2–4). Benson and Užgiris (2) demonstrated that infants who searched for a hidden object through self-initiated locomotion found it more frequently than infants who were passively transported to search locations suggesting that there is an association between self-directed independent mobility and spatial perception learning and memory. This concept is particularly important when developing technologies that enhance the ability of children with severe disabilities to participate in life activities through independent power mobility.

Children with developmental disabilities often have associated physical impairments, which result in limited independent self-directed exploration of their environment (5, 6). This limited self-initiated independent mobility and environmental exploration restricts learning and social participation, which research has shown can result in a cycle of decreased curiosity, social isolation, depression, and “learned helplessness” (7, 8). Furthermore, studies have reported that children with restricted independent mobility have lower rates of participation in life situations resulting in a perceived lower quality of life (9, 10).

Power mobility devices (PMD) provided to young children with mobility impairments have been shown to prevent some of these negative associated outcomes without causing deterioration of existing motor skills or interfering with development of new ones (11–15). Despite these reported positive effects, PMD is not typically recommended for children under the age of 3 years (16–18). Although there are many factors that affect power mobility use, such as social stigma of traditional powered mobility devices and parental/clinician/societal views of disability, the most common reason reported through a survey of power mobility prescribers for not recommending a PMD for young children with disabilities was no documentation of a successful power mobility trial required by funding sources (18). This is particularly true for children that need extended experience, such as those who have cognitive disabilities in addition to sensory-motor impairments (18).

One emerging power mobility device (PMD) option that has gained recognition as a means for a cost-effective trial of power mobility for young children is the battery powered modified ride-on toy car (19). Many groups, nationally and internationally, have been modifying battery powered ride-on toy cars by adding seating support and a large center placed push button switch for motor activation. These adapted ride-on toys are effective for teaching cause-and-effect but are limited in allowing children control over direction and driving velocity/acceleration (20). An activation control system that allows directional driving with speed control is the proportional joystick. A study conducted with children with significant cognitive and physical impairments found that the ability to understand cause-effect using a joystick to move a power wheelchair develops at an earlier age than understanding that pressing a single switch causes activation of wheelchair movements (21).

Traditionally children with severe physical and cognitive impairments are excluded from power mobility recommendations (22). If the child had additional sensory impairments such as visual, this exclusion was further reinforced (22). However, research has shown that these children are also capable of operating power mobility devices. Nilsson and Nyberg reported a case study of two children with profound cognitive disabilities and additional visual and motor impairments, who received training using a joystick-operated powered wheelchair (23). These children were 4 and 5 years of age and both were able to demonstrate intentional joystick activation after spending extended time in power wheelchairs.

It has been reported that only 40% of clinicians have access to loaner power wheelchairs for the necessary extended practice experiences (18). In addition, affordable, consistent access to a trial PMD is crucial for children with severe physical, visual and cognitive impairments to acquire essential skills needed for funding approval of a powered wheelchair. Modified ride-on toy cars may provide an interim solution to address these barriers and provide critical trial opportunities for these children. The purpose of this technical report is to (1) describe novel cost-effective proportional control joystick activation and steering modifications made to battery powered modified ride-on toy cars, and (2) provide evidence that very young children with severe multiple developmental impairments can learn to use this enhanced technology.

## Methods

The participants in this case series were seven children under the age of five with complex, severe disabilities. Local pediatric physical therapists working in rehabilitation outpatient facilities, early intervention programs, and public-school systems identified these children as needing access to an extended trial with a PMD to assess their potential for learning to use a power wheelchair. All the children had a primary diagnosis of cerebral palsy, significant cognitive impairments and were non-verbal. The individual's ability to follow directions and understand “cause and effect” relationships is presented in Table 1. Their Gross Motor Function Classification System (GMFCS) levels ranged between III and V. Six of the children had poor upper extremity control and four had cortical visual impairments.

**Table 1:** Participant characteristics and screening data.

**Participant number**	**Age in months**	**Gender**	**Health condition**	**Muscle tone**	**Active rom and strength**	**Spine alignment**	**GMFCS**	**Cognitive level**	**Seizures**	**Communication ability**	**Follows directions**	**Understands cause and effect**	**UE reaching**	**Vision**
2	36	Male	Cerebral palsy Quadriplegia	Spasticity	Decreased ROM UE and LE, L>R AFOs	Scoliosis, TLSO	IV	Delayed	No	Non-verbal uses Gestures	Emerging 75%	Yes	Poor/Dysmetria Profound impairment	Normal, corrected Glasses
3	48	Male	Cerebral palsy Quadriplegia VP shunt	Spasticity	Decreased in UE and LE AFOs Weakness in all extremities R>L	No scoliosis	V	Delayed, attends Exceptional Center based program for severe physical and cognitive disabilities	Yes	Non-verbal uses Gestures	No	Emerging	Poor/Dysmetria but does reach for toys	CVI
5	30	Male	Cerebral palsy Quadriplegia	Hypotonicity	ROM UE and LE Full; AFOs Weakness in all extremities	No scoliosis	IV	Significant Global Delay	Yes	Non-verbal uses Gestures	Yes	Yes	Poor/Dysmetria Trouble crossing midline when reaching for objects	CVI; Glasses
6	36	Female	Cerebral palsy	Spasticity	N/A	No Scoliosis	III	Delayed, attends Exceptional Center based program for severe physical and cognitive disabilities	Yes	Non-verbal uses Gestures	Emerging	Yes	Good	Normal, corrected Glasses
7	36	Male	Cerebral palsy Quadriplegia	Spasticity	Decreased ROM; Decreased strength L>R AFOs	N/A	V	Globally delayed ESE preschool	Yes	Non-verbal uses Gestures	Emerging	Emerging	Poor/Dysmetria; Reaches for toys	CVI; Glasses
8	12	Female	Cerebral palsy VP shunt	Hypotonia	ROM WNL Decreased strength	No scoliosis	IV	Globally Delayed	No	Non-verbal uses Gestures	Emerging	Emerging	Poor/Dysmetria reaches for objects	Normal, corrected Glasses
10	36	Male	Cerebral palsy Quadriplegia	Spasticity	ROM WNL AFOs and wrist splints	No Scoliosis	IV	Delayed ESE preschool	No	Non-verbal uses Gestures	Emerging	Emerging	Poor/Dysmetria; He reaches for objects	CVI

Each child had two visits over 3 months. During the first visit all required forms and consents were obtained as per the approved Institutional Review Board (IRB#6980116-11). An assessment similar to those done when prescribing a wheelchair seating system also took place. The ride on toy cars were designed and fabricated specifically for each child based on parental, therapist's goals and the child's needs. The second visit was dedicated to deliver the modified ride-on toy, and for family training/instruction.

### Modifications

Commercially available 12-V battery operated ride-on toy cars from Best Choice Products (Tustin, CA) were selected and modified. Most of the children/families that received these modified ride-on toys requested that they performed well in outdoor environments in the state of Florida, USA (e.g., grassy topography). Hence, 12-V ride-on toys were selected over 6-V due to their larger size and power, which allows them to operate indoors and outdoors, and over many terrains. The modifications were divided into two categories that addressed physiological, anatomical, and safety considerations: (1) seating and mechanical support (torso, upper/lower extremities, and neck/head), and (2) steering and activation mechanisms (electrical switching and drive system).

### Seating

Common seating and mechanical support modifications included raise seatbacks, head supports, chest straps, pelvic straps, pelvic lap belt, and lateral trunk supports pads. Easy to manipulate, safe, and off-the-shelf materials are typically used to keep these adaptations economically viable. The preferred materials consisted of Schedule 40 Polyvinyl Chloride (PVC) pipe (1″ diameter) for frame support and pool noodles, foam pads, or neoprene rubber for padding.

### Steering and Drive Adaptations

**Figure 1:**
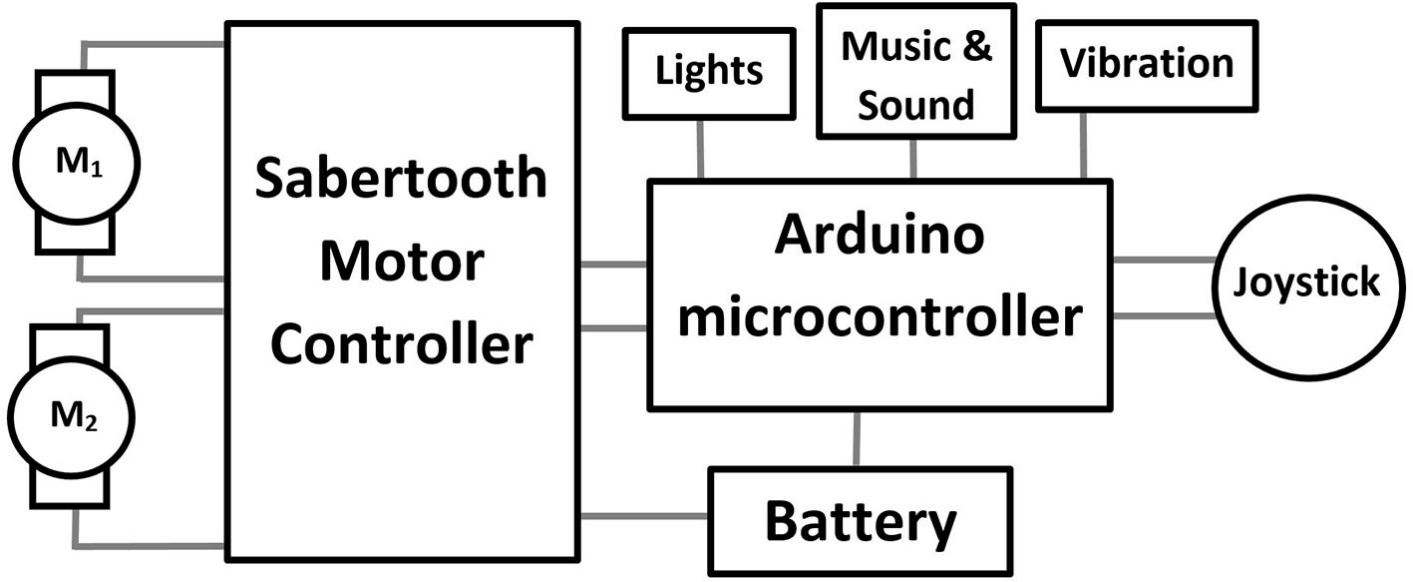
Block diagram of circuitry including joystick, microcontroller, motor controller and motors (M1 & M2).

Steering modifications, for enhanced control of the modified ride-on toys, were based on changes to the steering column and the implementation of an Arduino microcontroller (Somerville, MA) and a Sabertooth (Dimension Engineering, Hudson, OH) motor controller (Figure 1). The microcontroller was programmed to receive the signal from the joystick and communicate the desired action to the motor controller, which in turn provides proper voltage to the motors (M_1_ and M_2_). In addition, the Arduino had many other input output peripherals, which allowed the actuation of sensory activities that help engage the child with the toy, such as lights and music.

**Figure 2:**
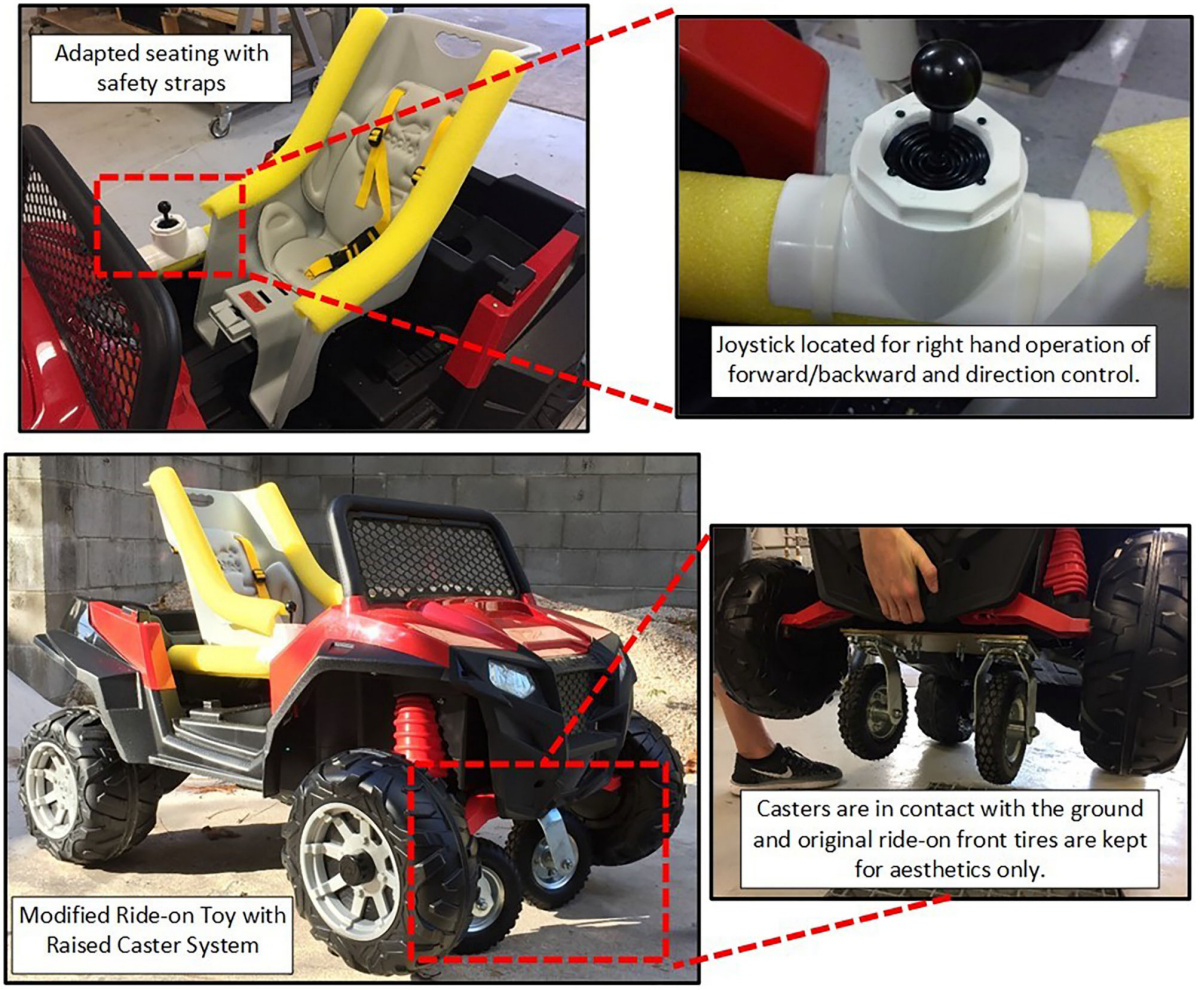
Modified ride-on toy car with seating, joystick and caster system.

There are two approaches that can be taken with these two motors. One consists of using M1 as the drive motor (i.e., forward and reverse) and M2 as the steering motor (left/right). The second approach, employed in this manuscript, consists of using the combination of both motors to simultaneously control drive and steering. This was accomplished by setting up a rear wheel drive system, with M1 controlling the left wheel and M2 controlling the right wheel. The front tires of the ride-on where replaced by casters. The activation mechanism was a proportional control joystick that allowed navigation of the car in any direction with enough precision to conduct a 3-point turn in 6 foot of space. If both motors were actuated equally the ride-on would move forward/backwards. If they were actuated unevenly, for example M1 on and M2 off, then the ride-on would turn. Furthermore, utilizing the Arduino microcontroller provided with the ability to limit the maximum speed of the ride-on and to add a ramping function that would avoid quick sudden or forceful movements once the motors were activated. Each ride-on was also programmed with a remote stop switch with override for parental control for emergency stopping. An example of these mechanical and electrical modifications to a ride-on toy car is shown in Figure 2. A summary of seating and steering modifications to each of the ride-on vehicles is presented in Table 2. It is noted that the mechanical and electrical adaptations presented are feasible for a Do-It-Yourself (DIY) enthusiast with minimal engineering skills. However, individuals interested in making these adaptations must become familiar with mechanical tools and processes (e.g., power tools for cutting and drilling), electrical tools and processes (e.g., soldering, wire gauge selection), and programming using the open source Arduino Integrated Development Environment (IDE).

**Table 2:** Description of seating and steering modifications per participant.

**Participant number**	**Diagnosis**	**Seating modifications**	**Steering modifications**
2	Cerebral palsy	• Increase back support by adding a foam board, seat hip angle 90° • H-strap harness seatbelt to prevent sliding forward or to the sides • Foam padding was added to the seat to increase comfort	• Proportional joystick was added to the right, the dominant side • Junction box was added to the right door to mount joystick • Foam padding added around junction box to cover sharp edges
3	Spastic quadriplegic cerebral palsy	• U-shaped back extended with foam covered PVC • Increase back support by adding a foam board, seat hip angle 90° • 4-point harness implemented	• Proportional joystick steering on the left door of the car • Joystick directly secured on the door of the ride-on with metal casing • Metal casing covered in foam
6	Cerebral palsy	• Back PVC structure for seatbelt attachment • Increase back support by adding a foam board, seat hip angle 90° • PVC structure covered in foam • H-shaped seatbelt attached from PVC structure to car	• Proportional joystick placed on right door of car • Joystick was placed inside junction box to encase exposed wires • Olaf head added to joystick to make appealing to child
5	Hypotonic quadriplegic cerebral palsy	• Strap seatbelt added to give some safety • Increase back support by adding a foam board, • seat hip angle 90°	• Proportional joystick added to front center of car • Center steering column section extended to create easier reach • Joystick added to end of extended column
7	Spastic quadriplegic cerebral palsy	• High seat back, head support • Chest strap • Pelvic belt • Lateral trunk supports • Abductor knee pads • Tilt in Space seat with seat 90° angle	• Proportional joystick placed on left side
8	Hypotonic quadriplegic cerebral palsy	• High seat back, head support • Chest strap • Pelvic belt • Seat hip angle 90°	• Joystick added to front center of car • Center steering column section extended to create easier reach
10	Spastic quadriplegic cerebral palsy	• High seat back, head support • Chest strap • Pelvic belt • Lateral trunk supports • Adductor hip pads • Tilt in Space seat with seat 90° angle	• Proportional joystick added to front center of car • Center steering column section extended to create easier reach

### Safety

It is important to note that all modifications described in this manuscript went through a two-fold process for safety assurance. First, an expert engineer conducted a safety inspection, which followed a standardized checklist approved by the Institutional Review Board. The checklist included visual inspections of the mechanical and electrical modifications to the ride-on and an operational check with 40 pounds at continuous speed for 10 min.

Second, the families were given a written care, safety and an operational manual as well as personal training in these areas. The families demonstrated the use and adjustment of harnesses, belts and correct positioning of their child in the car as well as the procedures for charging the battery and operation of the remote stop safety switch before the ride-on toys were provided for home use.

### Training

Every child was trained in the following manner: Once seated in the car, hand over hand guidance was used briefly to demonstrate to the child the “cause and effect” relationship between the joystick and car movement. Simple one-word verbal labels were used in conjunction with the haptic guidance, e.g., go, stop, and push. Once the joystick was demonstrated, the child was then given the opportunity for random, free exploration of the joystick, motor activation, and the consequent movement of the car without further adult instruction.

## Results

The participants were observed throughout the duration of their initial experience in their powered modified ride-on toy car. The initial experience lasted less than an hour for each child. All seven children were able to activate the modified ride-on car and experience the subsequent movement. Their activation became intentional and self-initiated. The participants would activate the car, stop and then repeat the activation. One interesting observation with the joystick operation was that all the children first pulled the joystick toward them, which initiated backward movement before learning to push the joystick to move the car forward.

A follow up phone call with the families occurred 3 months after the initial visit. It was reported by the caregivers that all the children were still using their PMD. The devices were being used for indoors and outdoors play with other children, for participation in family walks, and engagement of other children at school and on playgrounds. The only mechanical/electrical failure of the PMD reported was related to the battery not charging.

## Discussion

This case series technical report introduces modified ride-on toys with directional steering and programmable starting and driving velocity through a joystick, and the ability of children with severe multiple developmental impairments under the age of 5 years to operate them. The observational results of this study are consistent with other studies that have examined the ability of older children with multiple complex disabilities to learn to use a joystick to move a power wheelchair (24–27). In Nilsson's study the researchers noted that the participants demonstrated progressive behavior patterns in learning to use the joystick (23). These patterns were divided into 8 phases or levels of emerging competency in joystick use. Phase 1 indicated that the child has no awareness of using the joystick to move and phase 8 being functional use of the power wheelchair (23). Phase 4 is described as intentional activation of the joystick interspersed with self-initiated starts and stops in the movement (25).

In Nilsson's study, children having similar complex disabilities as the participants in this study were assessed at phase 1 at the completion of their initial experience (23). All seven of the children in this study achieved a phase 4 during their initial experience. One explanation for this difference in the phase levels of joystick use after initial experiences in these two groups may be the median age of the participants. The median age in this study was 3.7 years compared to a median age of 11 years in Nilsson's study (23). Perhaps examining current theories of sensorimotor development could offer some explanation for this difference.

For example, the dynamic systems theory and more recently the Neuronal Group Selection theory propose the brain goes through a critical period of development that serves as the underpinning for future cognitive and sensorimotor function (28, 29). This critical period occurs before the age of three (29). Therefore, opportunities for self-initiated sensorimotor interactions with the environment at very young ages support optimal motor learning (29). This may explain the higher level of joystick use attained with the initial experience in younger children in the current study.

### Clinical Implications

One economical way to provide cause-effect learning opportunities in preparation for beginning power mobility use by children with complex multiple impairments is the provision of modified ride-on toy cars as previously reported in the literature (20). The observational case presented in this manuscript suggests that by replacing the typical push button activation switch with a proportional joystick to provide easier sustained activation and directional control may prove to be an effective, economical solution to provide extended practice time for learning independent, self-directed power mobility to very young children. The total average cost of the joystick adaptations per PMD presented in this manuscript was ~$540.00 per PMD. This includes average cost of a ride-on toy bought in 2018 ($250), Sabertooth motor controller with dealer discount ($85, $120 without dealer discount), Arduino UNO ($18 for 2-unit kit), and about $200 in supplies such as foam, PVC, bolts, etc. The cost does not include power and hand tools employed for modifying the ride-on toys. This amount is significantly less than the average cost of $7,132.00 for a power wheelchair with adjustable speed joystick reported by Rentschler et al. (30).

The observations in the current study, while supportive of previous investigations, offers new information for power mobility candidate evaluation. Children 5 years and younger with severe and profound complex disabilities at early developmental levels were able to self-initiate movement using a joystick interface in a short period of time through free exploration without specific training. All seven children are part of an ongoing investigation to determine if prolonged practice in the joystick modified ride-on toy cars translates into skill acquisition that would be consistent with traditional power wheelchair readiness assessments.

## Data Availability Statement

All datasets generated for this study are included in the article/supplementary material.

## Ethics Statement

The studies involving human participants were reviewed and approved by University of North Florida Institutional Review Board (IRB) IRB#6980116-11: The Impact of Technology Assisted Toy Play on the Independent Function and Quality of Life in Preschool Children with Disabilities. Written informed consent to participate in this study was provided by the participants' legal guardian/next of kin.

## Author Contributions

JA led the mechanical and electrical modifications and carried out all safety testing. ML performed, and analyzed data from all children. JA and ML wrote and edited the manuscript. All authors contributed to the article and approved the submitted version.

## Conflict of Interest

The authors declare that the research was conducted in the absence of any commercial or financial relationships that could be construed as a potential conflict of interest.
